# Physiologically based pharmacokinetic modeling of fluconazole: interpretation of gender-dependent pharmacokinetics and cytochrome P450 inhibition

**DOI:** 10.3389/fphar.2026.1849886

**Published:** 2026-07-29

**Authors:** Kazuya Ishida, Rakshit Sanjay Tanna, Jesse Yang, Yurong Lai

**Affiliations:** Drug Metabolism, Gilead Sciences, Inc., Foster, CA, United States

**Keywords:** cytochrome P450, drug–drug interactions, fluconazole, physiologically-based pharmacokinetic model, volume of distribution

## Abstract

The accurate prediction of clinical drug–drug interactions (DDIs) in modeling analysis relies on the fraction of metabolism and transport of the substrate drugs and on the inhibitory potency and plasma exposure of index inhibitors. Fluconazole is frequently used as a clinical index inhibitor of cytochrome P450 (CYP) 2C9 and CYP3A4 to assess DDI liabilities involving these CYP enzymes. It also inhibits uridine diphosphate glucuronosyltransferase (UGT) 2B7 and exhibits gender-dependent pharmacokinetics (PK). The currently available model profile of fluconazole does not account for these population-specific PK variations. Therefore, extrapolating from one fluconazole DDI dataset to another remains questionable when it is used as an index inhibitor. In the present study, a physiologically based pharmacokinetic (PBPK) model of fluconazole was developed using PK data from male and female subjects. A minimal PBPK model with advanced dissolution, absorption, and metabolism (ADAM) for fluconazole was built in the Simcyp Simulator (V22). The base model from the Simcyp compound library captured fluconazole plasma profiles following oral administration only in male subjects, but not in female subjects. The volume of distribution at steady state (V_ss_) of fluconazole is close to the total body water volume, which can contribute to differences in plasma profiles between male and female subjects. Therefore, V_ss_ in female subjects was optimized by curve fitting. The total clearance (CL) value in female subjects was also adjusted to reflect the similar fluconazole elimination half-life (t_1/2_) between male and female subjects. The plasma profiles of fluconazole in the general population were captured well when the predicted mean V_ss_ and total CL were calibrated by the proportion of female subjects. The developed model was further validated by simulating reported clinical DDI studies with fluconazole. Using the refined PBPK model, the inhibitory constants (Ki) for fluconazole against CYP2C9 and UGT2B7 were optimized. The final fluconazole model captured the precipitant DDIs with midazolam (CYP3A probe substrate), tolbutamide (CYP2C9 probe substrate), omeprazole (CYP2C19 probe substrate), and zidovudine (UGT2B7 probe substrate), indicating that the refined fluconazole PBPK model in the present study can accurately predict clinical DDIs across studies in different populations with diverse gender mixes.

## 1 Introduction

Drug–drug interaction (DDI) occurs when a substrate drug is coadministered with another drug that acts as an inhibitor or inducer. Depending on the contribution of such metabolizing enzymes and transporters to the substrate drugs, the interactions can change plasma levels, potentially resulting in clinically significant effects, labeling restrictions, or unfavorable outcomes of the drug (Neuvonen et al., 1998; Niemi et al., 2003). For example, plasma exposure of simvastatin (40 mg single dose on day 4), an HMG-CoA reductase inhibitor, was increased 19-fold in both the area under the plasma concentration–time curve (AUC) to infinity (AUC_inf_) and maximum concentration (C_max_) in healthy volunteers by itraconazole (200 mg daily for 4 days), a well-known cytochrome P450 (CYP) 3A4 inhibitor (Neuvonen et al., 1998). The complete inhibition of CYP3A by itraconazole led to a tremendous increase in simvastatin exposure, leading to an increase in the potential risk of adverse effects, such as rhabdomyolysis. Niemi et al. (2003) evaluated the possible DDI of gemfibrozil, a fibrate drug to manage hypertriglyceridemia, with repaglinide, which is used to lower blood sugar in type 2 diabetes, and found that the AUC_inf_ and C_max_ of repaglinide (0.25 mg single dose on day 3) were increased 8.1- and 2.4-fold, respectively, by gemfibrozil coadministration (600 mg twice daily for 3 days). The higher plasma exposure of repaglinide by gemfibrozil may increase the risk of hypoglycemia. Repaglinide is a substrate of CYP2C8 and OATP1B1; dual inhibitory mechanisms can explain the greater DDI effects between repaglinide and gemfibrozil. These examples underscore the importance of identifying precise DDI mechanisms to avoid the undesired drug effects.

Fluconazole is a bis-triazole derivative developed for the treatment of superficial and systemic fungal infections, which predominantly affect immunocompromised individuals (Debruyne and Ryckelynck, 1993). It is a potent CYP2C9 inhibitor with an *in vitro* inhibition constant (Ki) value of 7–8 µM (Kunze et al., 1996) and can moderately inhibit CYP2C9 at clinically relevant doses. As a potent CYP2C19 inhibitor (Ki = 2 μM) (Wienkers et al., 1996), fluconazole could enhance the effects of proton pump inhibitors such as omeprazole (Kang et al., 2002). Fluconazole is also a moderate inhibitor of CYP3A (Gibbs et al., 1999), with inhibitory effects weaker than those of itraconazole. Fluconazole has been shown to elevate exposure to statins, benzodiazepines, calcium channel blockers, and immunosuppressants, thereby raising risks such as myopathy, sedation, hypotension, and toxicity (Canafax et al., 1991; Hylton Gravatt et al., 2017; Kremens et al., 1999; Ulivelli et al., 2004). Additionally, it inhibits CYP3A5 (Gibbs et al., 1999), though to a lesser extent than CYP3A4, contributing to variability in drug metabolism. Fluconazole also inhibits uridine diphosphate glucuronosyltransferase (UGT) 2B7 (Uchaipichat et al., 2006) and increases the plasma concentration of zidovudine, a UGT2B7 probe substrate (Sahai et al., 1994). As such, fluconazole has been included in the International Council for Harmonisation of Technical Requirements for Pharmaceuticals for Human Use (ICH) M12 guidance on drug interaction studies as a clinical index inhibitor for CYP2C9 (moderate) and CYP2C19 (strong) and is noted as a moderate inhibitor for CYP3A (ICH, 2024).

Fluconazole is a weak base, with an acid dissociation constant (pKa) of 1.76, which allows it to remain mostly unionized at intestinal pH, facilitating passive diffusion across the intestinal epithelium. Oral absorption of fluconazole is relatively fast with oral bioavailability exceeding 90% (Debruyne and Ryckelynck, 1993). Fluconazole exhibits moderate plasma protein binding (11%–12% free). Approximately 80% of the dose is primarily eliminated unchanged via renal excretion, and approximately 11% is metabolized to fluconazole glucuronide. The elimination half-life (t_1/2_) of fluconazole is relatively long, approximately 30 h in adults with normal renal function (Debruyne and Ryckelynck, 1993). Interestingly, a controlled clinical trial in healthy volunteers demonstrated gender-dependent plasma pharmacokinetic (PK) profiles (Carrasco-Portugal and Flores-Murrieta, 2007), whereas the apparent volume of distribution following oral administration of fluconazole (Vd/F) in the female population (0.623 L/kg) was substantially lower than that in the male population (0.751 L/kg). It was concluded by the authors that the difference in Vd/F between male and female subjects is probably due to the difference in their respective total body water volume (Carrasco-Portugal and Flores-Murrieta, 2007).

Fluconazole is a readily soluble and highly permeable molecule, a Class I drug based on the Biopharmaceutical Classification System. It shows gender-dependent PK, which could influence the extent of its DDIs with substrate drugs across different populations with diverse gender mixes. It is generally an established practice to use data from clinical DDI studies involving index inhibitors or inducers to determine the fraction of metabolism (f_m_) contributed by a particular enzyme, e.g., CYP2C9. Once f_m_ is determined, it is often combined with *in vitro* and *in vivo* data using a physiologically based PK (PBPK) modeling approach to simulate untested DDI scenarios, assess the effects of genetic polymorphisms, and evaluate the impact on special populations, ultimately to inform drug labels. When gender or age differences affect inhibitor PK parameters, it is crucial to first develop population-specific PK profiles and then use population-matching approaches for model simulations. Therefore, developing a model that captures potential differences in the magnitude of fluconazole DDI between male and female subjects is necessary for DDI evaluations when fluconazole is used as an index inhibitor. Additionally, these effects are dose dependent, becoming more evident at doses of ≥200 mg/day, and may persist because of fluconazole’s long elimination half-life, requiring careful monitoring when combined with substrate drugs (Lazar and Wilner, 1990). In this study, a PBPK model of fluconazole is developed to capture its plasma profiles in male-only, female-only, and mixed male and female populations. Additionally, DDI simulations between fluconazole (precipitant) and relevant probe substrates (objects) were conducted to validate this model and assess the inhibitory effect of fluconazole across different gender compositions.

## 2 Methods

### 2.1 Human PK data collection

The human plasma concentration profiles of fluconazole were obtained from the literature and included in the current modeling analysis. Table 1 lists the studies utilized in this study. To validate the developed PBPK model, literature DDI data between fluconazole (precipitant) and substrate drugs, including midazolam (CYP3A probe substrate), tolbutamide (CYP2C9 probe substrate), *S*-warfarin (CYP2C9 probe substrate), omeprazole (CYP2C19 probe substrate), and zidovudine (UGT2B7 probe substrate) were also used. Table 1 also summarizes the study information.

**TABLE 1:** Human PK reported in the literature used for model development and validation.

Compound	Dosing regimen	Subjects				References
		Population	No. of subjects	Age (years)	% female	
Fluconazole	100 mg SD	HV	14	21–29	0%	Thorpe et al. (1990)
	400 mg SD	HV	14	19–55	100%	Adedoyin et al. (2021)
	50 mg SD	HV	20	20–65	60%	Toon et al. (1990)
	100 mg SD	HV	24	18–49	42%	Zimmermann et al. (1994)
	200 mg SD	HV	16	18–45	0%	Apseloff et al. (1991)
	400 mg SD	HV	9	19–25	44%	Ahonen et al. (1997)
	100 mg SD	HV	26	20–50	0%	Carrasco-Portugal and Flores-Murrieta (2007)
	100 mg SD	HV	33	20–50	100%	
	50 mg QD	HV	8	20–32	62.5%	Varhe et al. (2003)
	100 mg QD	HV	8	20–32	62.5%	
	400 mg LD + 200 mg QD	HV	8	20–32	62.5%	
Midazolam	SD of 400 mg fluconazole orally and then SD of 7.5 mg midazolam 2 h after fluconazole administration	HV	12	19–25	42%	Olkkola et al. (1996)
	SD of 400 mg fluconazole orally and then SD of 1 mg midazolam administered intravenously 2 h after fluconazole administration	HV, CYP3A5 *1/*1 (expressor)	6	18–45	50%	Isoherranen et al. (2008)
		HV, CYP3A5 *1/*X	7	18–45	71.4%	
		HV, CYP3A5 *X/*X (non expressor)	6	18–45	83.3%	
Tolbutamide	SD of 150 mg fluconazole orally and then SD of 500 mg tolbutamide orally 2 h after fluconazole administration	HV	13	20–50	0%	Lazar and Wilner (1990)
*S*-warfarin	400 mg QD fluconazole orally for 21 days; on day 14, SD of 8.75 mg *S*-warfarin was coadministered orally	HV, CYP2C9 *1/*1 only	10	21–52	10%	Bavisotto et al. (2011)
	400 mg QD fluconazole orally for 14 days; on day 8, SD of 0.375 mg/kg *S*-warfarin was administered orally 1 h before fluconazole administration	HV	6	23–39	0%	Black et al. (1996)
Omeprazole	400 mg QD fluconazole orally for 5 days; on day 5, SD of 20 mg omeprazole was coadministered orally	HV	18	23–28	0%	Kang et al. (2002)
	50 mg QD fluconazole administered orally for 9 days; on day 9, SD of 20 mg omeprazole was coadministered orally	HV	6	22–36	0%	Park et al. (2017)
Zidovudine	400 mg QD fluconazole orally for 7 days; on day 7, SD of 200 mg zidovudine was coadministered orally	Subjects who are HIV-positive	12	23–51	0%	Sahai et al. (1994)

HIV: human immunodeficiency virus. HV, healthy volunteers; LD, loading dose; QD, once daily; SD, single dose; CYP, cytochrome P450.

### 2.2 PBPK model analysis

**FIGURE 1:**
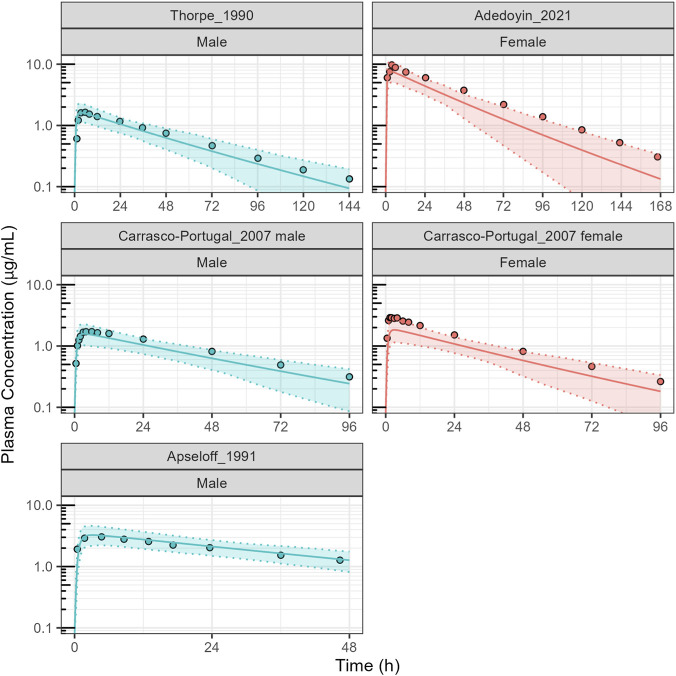
Observed versus simulated plasma profiles of fluconazole using the base physiologically based pharmacokinetic (PBPK) model in male-only and female-only subjects. The base PBPK model from the Simcyp compound library was used for simulation. The simulations were repeated 10 times. The closed circles represent the observed plasma concentration. The solid lines represent the mean of the virtual population. The shaded areas represent the 5th–95th percentiles of the total virtual population.

A minimum PBPK model with the advanced dissolution, absorption, and metabolism (ADAM) model for fluconazole was developed using the Simcyp Simulator® version 22 (Certara, Sheffield, United Kingdom). The volume of distribution at steady state (V_ss_) of fluconazole in female subjects was estimated using the parameter estimation function in the Simcyp Simulator, based on fluconazole plasma profiles from only female subjects (Adedoyin et al., 2021). After estimating V_ss_ in female subjects, the *in vivo* total clearance (CL) of fluconazole was optimized to match the mean t_1/2_ of the drug from two studies (28.2 and 33.6 h) (Adedoyin et al., 2021; Carrasco-Portugal and Flores-Murrieta, 2007). Moreover, the renal CL (CL_R_) of fluconazole was adjusted to maintain a similar percentage of renal elimination (∼80%). Since the plasma profile of fluconazole in male subjects were captured well (see Figure 1), the Simcyp default values of CL and V_ss_ were applied to male subjects (Table 2). Fluconazole plasma profiles in mixed male and female populations were simulated using calibrated V_ss_ and CL values. The calibrated V_ss_ and CL values were obtained from the following equations:

$$V_{ss}^{calibrated}=V_{ss}^{male}\cdot \left(1-P_{female}\right)+V_{ss}^{female}\cdot P_{female},$$ (1)

$${CL}^{calibrated}={CL}^{male}\cdot \left(1-P_{female}\right)+{CL}^{female}\cdot P_{female},$$ (2)

where $V_{ss}^{male}$ and $V_{ss}^{female}$ are the mean V_ss_ in male and female subjects (0.76 and 0.51 L/kg), respectively. ${CL}^{male}$ and ${CL}^{female}$ are the mean CL in male and female subjects (1.21 and 0.78 L/h), respectively $.\;P_{female}$ is the proportion of female subjects in each study. The final input parameters for fluconazole are listed in Table 2.

**TABLE 2:** Input parameters of the fluconazole PBPK model.

Parameter	Value	Source
Physicochemical property		
Molecular weight (g/mol)	306.3	Simcyp
Log P	0.2	Simcyp
Compound type	Monoprotic base	​
pKa	1.76	Simcyp
f_u,plasma_	0.89	Simcyp
Absorption—ADAM model		
f_u,gut_	0.89	Simcyp
P_eff,man_	4.14	Simcyp
P_trans,0_	29.257	Simcyp
Distribution—minimal PBPK model		
V_ss_ (L/kg)	0.76 (male) 0.51 (female)	Male: Simcyp Female: Optimized
Elimination—in vivo clearance		
CL (L/h)	1.21 (male) 0.78 (female)	Male: Simcyp Female: Optimized
CL_R_ (L/h)	0.86 (male) 0.554 (female)	Male: Simcyp Female: Calculated (keep the same CL:CL_R_ ratio)
Interaction		
CYP2C9 Ki (μM)	8.5	Optimized
CYP2C9 fu_mic_	0.89	Simcyp
CYP2C19 Ki (μM)	2	Simcyp
CYP2C19 fu_mic_	1	
CYP3A4 Ki (μM)	10.7	Simcyp
CYP3A4 fu_mic_	1	
CYP3A5 Ki (μM)	84.6	Simcyp
CYP3A5 fu_mic_	1	
UGT2B7 Ki (μM)	30	Optimized
UGT2B7 fu_mic_	1	Simcyp

ADAM, advanced dissolution, absorption and metabolism; CL, total clearance; CLR: renal clearance; PBPK, physiologically-based pharmacokinetic.

The PBPK models for midazolam, tolbutamide, *S*-warfarin, and omeprazole were obtained from the Simcyp compound library. Since zidovudine’s plasma profile at the absorption phase (up to 3 h) was not captured well using the Simcyp compound library file, the absorption of zidovudine was optimized using observed plasma profiles of the drug obtained from the literature. The detailed model was described in the Supplementary Material. The Ki of fluconazole for CYP2C9 and UGT2B7 was refined using observed DDI data (CYP2C9, fluconazole–tolbutamide DDI; UGT2B7, fluconazole–zidovudine DDI, listed in Table 1). The acceptance criteria for the DDI simulation were set so that the simulated/observed AUC and C_max_ ratio was within 0.8–1.25.

## 3 Results

### 3.1 Fluconazole plasma profiles simulation using the base and refined PBPK model

The PBPK model of fluconazole in the Simcyp compound library was first used as a base model to simulate the plasma profile in male- and female-only subjects (Figure 1). The base model captured well the plasma profile of fluconazole in male-only subjects; however, the simulated plasma concentration of fluconazole in female-only subjects was underestimated (Figure 1).

**FIGURE 2:**
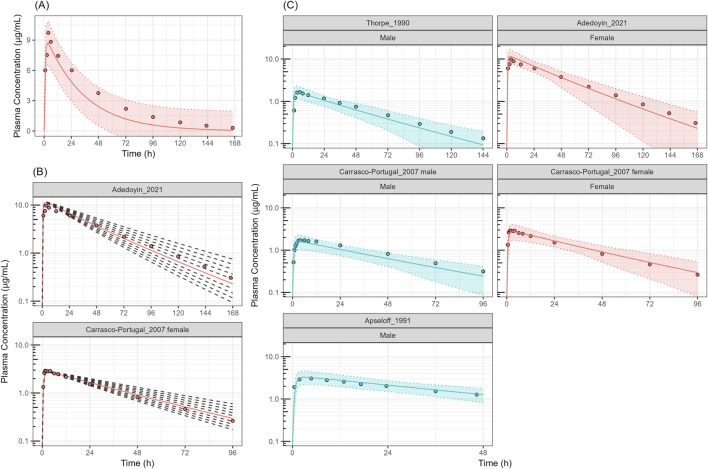
Fitted and simulated plasma profiles of fluconazole in male-only and female-only subjects. **(A)** Volume of distribution at steady state (V_ss_) in female subjects was from observed data 0.51 L/kg (90% CI: 0.45–0.58). The solid lines represent the predicted mean concentration. The shaded areas represent the prediction interval computed by the Simcyp Simulator. **(B)** The sensitivity analysis of clearance (CL) in female subjects. The total CL was further refined to 0.78 L/h (solid red line) using the measured plasma t_1/2_ in female subjects (mean t_1/2_: 30.9 h). The black dashed lines represent the simulated concentration with CL 0.53–0.98 L/h. **(C)** The simulations using a refined model were repeated 10 times. The closed circles represent the observed plasma concentration. The solid lines represent the mean concentration of the virtual population. The shaded areas represent the 5th–95th percentiles of the total virtual population.

The Vd/F of fluconazole in female subjects was reported to be lower than that in male subjects, likely due to differences in total body water volume (Carrasco-Portugal and Flores-Murrieta, 2007). V_ss_ in the base model was fitted to the PK data observed in female subjects, 0.51 L/kg (90% CI: 0.45–0.58) (Figure 2A). As the t_1/2_ of fluconazole is reported to be ∼30 h in both male and female subjects (Debruyne and Ryckelynck, 1993), the CL in female subjects (0.78 L/h) was optimized to capture the reported t_1/2_ in female subjects (mean value from two studies: 30.9 h). CL_R_ in the female population (0.554 L/h) was also adjusted to maintain the same renal drug elimination rate (∼80%). The plasma profiles of fluconazole in the female-only subjects were captured well after incorporating the estimated V_ss_ and the adjusted CL/CL_R_ (Figure 2B).

**FIGURE 3:**
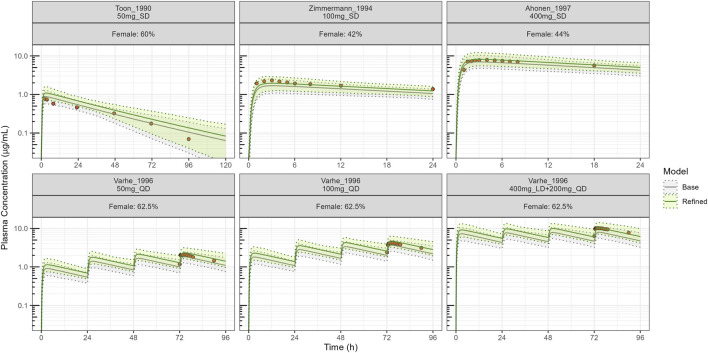
Observed versus simulated plasma profiles of fluconazole using refined physiologically based pharmacokinetic (PBPK) model in mixed male and female subjects. Volume of distribution at steady state (V_ss_) and total clearance (CL) of fluconazole were calibrated using Equations 1, 2 in each study (see Section 2). The simulations were repeated 10 times. The closed circles represent the observed plasma concentration. The solid lines represent the mean concentration of the virtual population. The shaded areas represent the 5th–95th percentiles of the total virtual population.

The plasma profiles of fluconazole in mixed male and female subjects were simulated after calibrating V_ss_ and CL (Equations 1 and 2; see Section 2), and the simulated curves were overlaid on the observed profiles (Figure 3).

### 3.2 Object model optimization

The Simcyp Simulator compound library models for midazolam, tolbutamide, *S*-warfarin, and omeprazole captured well the observed data in the literature (data not shown). However, zidovudine plasma profiles, especially the absorption phase, were underpredicted by the Simcyp compound library model. To capture zidovudine absorption, the absorption rate scalar was optimized (Supplementary Table S2). The refined zidovudine model captured the plasma profiles of the drug and its major metabolite, zidovudine glucuronide.

### 3.3 DDI simulation

Using the refined PBPK models, DDIs between fluconazole (precipitant) and probe substrates of CYP and UGT enzymes were simulated. The CYP2C9 Ki value for fluconazole was optimized to 8.5 μM based on the fluconazole–tolbutamide DDI (Table 3), and the fluconazole PBPK model with this optimized CYP2C9 Ki value successfully predicted another CYP2C9-mediated DDI, fluconazole–*S*-warfarin DDI (Table 3). The UGT2B7 Ki value was adjusted to 30 μM to accurately simulate the fluconazole–zidovudine DDI (Table 3).

**TABLE 3:** Observed and predicted mean AUC and C_max_ ratios for midazolam, tolbutamide, omeprazole, and zidovudine.

Object	Dosing regimen	Target enzyme	Observed		Predicted		Pred/Obs		References
			AUC	C_max_	AUC	C_max_	AUC	C_max_	
Midazolam	SD of 400 mg fluconazole orally and then SD of 7.5 mg midazolam 2 h after fluconazole administration	CYP3A	3.51	2.50	3.33	2.00	0.95	0.98	Olkkola et al. (1996)
	SD of 400 mg fluconazole orally and then SD of 1 mg midazolam administered intravenously 2 h after fluconazole administration	CYP3A (CYP3A5 *1/*1 only)	1.60	NA	1.68	NA	1.05	NA	Isoherranen et al. (2008)
		CYP3A (CYP3A5 *1/*X only^a^)	1.65	NA	1.77	NA	1.07	NA	
		CYP3A (CYP3A5 *X/*X only^b^)	1.95	NA	1.92	NA	0.985	NA	
Tolbutamide	SD of 150 mg fluconazole orally and then SD of 500 mg tolbutamide orally 2 h after fluconazole administration	CYP2C9	1.43	1.09	1.74	1.16	1.22	1.06	Lazar and Wilner (1990)
*S*-warfarin	400 mg QD fluconazole orally for 21 days; on day 14, SD of 8.75 mg *S*-warfarin was coadministered orally		2.56^c^	1.15^c^	2.36^c^	1.05^c^	0.922	0.913	Bavisotto et al. (2011)
	400 mg QD fluconazole orally for 14 days; on day 8, SD of 0.375 mg/kg *S*-warfarin was administered orally 1 h before fluconazole administration		2.84	NR	3.44	1.07	1.21	NA	Black et al. (1996)
Omeprazole	400 mg QD fluconazole orally for 5 days; on day 5, SD of 20 mg omeprazole was coadministered orally	CYP2C19	6.29	2.40	6.80	2.80	1.08	1.17	Kang et al. (2002)
	50 mg QD fluconazole administered orally for 9 days; on day 9, SD of 20 mg omeprazole was coadministered orally		4.33^c^	2.68^c^	3.84^c^	2.20^c^	0.887	0.821	Park et al. (2017)
Zidovudine	400 mg QD fluconazole orally for 7 days; on day 7, SD of 200 mg zidovudine was coadministered orally	UGT2B7	1.74	1.84	1.96	1.55	1.13	0.842	Sahai et al. (1994)

AUC, area under the plasma concentration–time curve; C_max_, maximum concentration; CYP, cytochrome P450; UGT, uridine diphosphate glucuronosyltransferase; HVs, healthy volunteers; LD, loading dose; NA, not applicable; NR, not reported; obs, observed; pred, predicted; QD, once daily; SD, single dose.

^a^ CYP3A5 *1/*X includes CYP3A5 *1/*3 and *1/*6.

^b^ CYP3A5 *X/*X includes CYP3A5 *3/*3 and *3/*7.

^c^ Geometric mean ratio.

## 4 Discussion

Male subjects generally have higher total body, extracellular, and intracellular water and total plasma and red blood cell volumes than female subjects, primarily because of lower body fat and higher lean body mass. Consequently, this variation in body composition can affect the PK of water-soluble drugs, often leading to increased V_ss_ in male subjects (Mauvais-Jarvis et al., 2021; Vahl et al., 1997). For example, ofloxacin shows notably lower Vd/F (25% reduction) in female subjects compared with that in male subjects, which can be attributed to gender-related differences in body weight (Sowinski et al., 1999). Similarly, the V_ss_ of other compounds, such as levofloxacin and ethanol, also differed between male and female subjects due to the difference in total body water volume (Overholser et al., 2005; Soldin and Mattison, 2009). As the average V_ss_ of fluconazole is similar to the total body water volume (∼0.7 L/kg) in male subjects, the plasma exposure difference between male and female subjects can be explained by the differences in total body water volume (Carrasco-Portugal and Flores-Murrieta, 2007). As such, the PK difference between male and female subjects can be an issue for DDI risk assessment when fluconazole is used as an index inhibitor in clinical DDI trials, when the data are used to extrapolate the untested scenarios in populations with different gender compositions. Thus, gender-related PK variability needs to be considered when building an inhibitor PK model for DDI prediction. In this study, the observed V_ss_ was used in female subjects (0.51 L/kg) (Adedoyin et al., 2021), which is comparable with that reported by Carrasco-Portugal and Flores-Murrieta (2007), considering ∼90% oral bioavailability of the drug (Debruyne and Ryckelynck, 1993). Additionally, the glomerular filtration in male subjects is consistently higher than that in female subjects (Schwarz, 2003). Although glomerular filtration is directly proportional to body weight, there may be weight-independent gender differences in the glomerular filtration rate (GFR) (the measured GFR in male and female subjects was 114.8 and 105.9 mL/min/1.73 m^2^, respectively) (Gross et al., 1992). Additionally, the t_1/2_ of fluconazole in male subjects was reported to be 32–35 h (Apseloff et al., 1991; Carrasco-Portugal and Flores-Murrieta, 2007; Thorpe et al., 1990), whereas that in female subjects was 28–33 h (Adedoyin et al., 2021; Carrasco-Portugal and Flores-Murrieta, 2007). The CL in female subjects (0.78 L/h) was optimized to achieve the observed t_1/2_. The refined PBPK model of fluconazole, with optimized V_ss_ and CL in female subjects, captured the plasma profiles (Figure 2). Additionally, in mixed male and female populations, the simulated plasma profiles of fluconazole by the refined PBPK model (calibrated for V_ss_ and CL by the proportion of female subjects using Equations 1 and 2) were closer to observed data compared with the simulated curves by the base PBPK model (Figure 3).

To evaluate the inhibitory effect of fluconazole on CYP3A, CYP2C9, CYP2C19, and UGT2B7, we assessed whether the PBPK model of the probe substrate in the Simcyp compound library captures the plasma profiles of the substrates. The plasma profiles of midazolam (CYP3A probe substrate), tolbutamide (CYP2C9 probe substrate), *S*-warfarin (CYP2C9 probe substrate), and omeprazole (CYP2C19 probe substrate) were captured well using the models in the Simcyp compound library. However, the plasma profile of zidovudine (a UGT2B7 probe substrate) was captured poorly, especially in the absorption phase. Zidovudine is a nucleoside analog and is a substrate of concentrative nucleoside transporter (CNT) 1 and CNT3, which are expressed in the gut (Pastor-Anglada et al., 2018; Yang and Xu, 2022). Additionally, zidovudine efflux from primary human endothelial cells obtained from the brain and aorta was remarkably increased by MK-571 [a well-known inhibitor of multidrug resistance-associated proteins (MRPs)] but not by verapamil (a P-glycoprotein inhibitor), suggesting that zidovudine is also a substrate of MRPs (Eilers et al., 2008). Therefore, zidovudine absorption may be mediated not only by passive diffusion but also by active transport processes. To capture this active process, we optimized absorption rate scalars for zidovudine (Supplementary Table S2), and the refined PBPK model of zidovudine captured its plasma profile and that of its major metabolite, zidovudine 5′-*O*-glucuronide (Supplementary Figure S1).

Using the refined PBPK models, DDI simulations were conducted with default Ki values for fluconazole with CYP3A4 and CYP3A5; the models captured changes in the AUC and C_max_ of midazolam upon coadministration of fluconazole. Isoherranen et al. (2008) reported midazolam–fluconazole DDI in healthy volunteers, and enrolled subjects were grouped by CYP3A5 genotype. The refined PBPK model accurately captured the AUC ratio of midazolam following IV administration in all three groups, supporting the validity of the Ki values of fluconazole for both CYP3A4 and CYP3A5. In contrast, the Ki value of fluconazole for CYP2C9 needs to be optimized using tolbutamide–fluconazole DDI data (Lazar and Wilner, 1990), yielding the optimized Ki value of 8.5 μM (Table 2). The optimized Ki value was able to simulate another CYP2C9 substrate (*S*-warfarin)–fluconazole DDI (Table 3).

The investigation has several limitations. In general, DDI studies are conducted in healthy volunteers; therefore, all simulations were conducted in healthy volunteers (except zidovudine DDI simulations, no plasma profile of the drug is available in healthy volunteers). However, extra caution may be required when the final fluconazole PBPK model is used to simulate the DDI potential in patients. For example, the CL:CL_R_ ratio was assumed to be the same between male and female subjects. However, this ratio may be different between male and female subjects due to GFR differences. Since fluconazole is mainly excreted into the urine, the additional calibration of CL in female patients with renal impairment may be required. Additionally, total body water is changed by renal function (Chumlea et al., 2001). Therefore, a different V_ss_ calibration in patients with renal impairment may be required. The Ki value of fluconazole for UGT2B7 was optimized using zidovudine–fluconazole DDI data (Sahai et al., 1994). Since no study data are available for other UGT2B7–fluconazole DDIs, further studies are warranted for the calibration of Ki values for UGT2B7.

In conclusion, we developed and validated a fluconazole PBPK model that captures plasma profiles in both male and female subjects. Additionally, the Ki values of fluconazole for CYP2C9 and UGT2B7 were optimized to capture the reported DDIs. The refined model can be used to simulate potential scenarios with fluconazole coadministration prospectively in both male and female subjects. Furthermore, the gender-specific model in this study can be useful for different populations to understand DDI mechanisms, especially CYP3A- and CYP2C9/19-mediated metabolism, before conducting clinical trials by using fluconazole as an index inhibitor.

## Data Availability

The original contributions presented in the study are included in the article/Supplementary Material; further inquiries can be directed to the corresponding author.
