# Supplementary material for: Physiologically based pharmacokinetic modeling of fluconazole: interpretation of gender-dependent pharmacokinetics and cytochrome P450 inhibition
Source: Front Pharmacol. 2026 Jul 29;17:1849886. doi: 10.3389/fphar.2026.1849886 (PMC13462044; doi:10.3389/fphar.2026.1849886)
Supplement: Supplementary file 1 [file DataSheet1.pdf]

Physiologically-Based Pharmacokinetic Modeling of Fluconazole: Interpretation of Gender-Dependent Pharmacokinetics and Cytochrome P450 Inhibition

Kazuya Ishida, Rakshit Sanjay Tanna, Jesse Yang, Yurong Lai

Supplementary Material

Supplemental Methods

Development of Zidovudine and Zidovudine 5’-*O*-Glucuronide PBPK Model

A full PBPK model with ADAM function for zidovudine was developed using Simcyp Simulator® version 22 (Certara, Sheffield, UK). The observed plasma profiles of zidovudine and its major metabolite, zidovudine 5’-*O*-glucuronide (if available) were obtained from the literature (**Supplemental Table 1**). The physicochemical properties of the drug, drug distribution, and elimination data were collected from Simcyp compound library. To capture the plasma profile of zidovudine at the absorption phase (up to 3 h after the dose), the absorption rate scalars and basolateral absorption scalars were optimized. The input parameters of zidovudine and zidovudine 5’-*O*-glucuronide are listed in **Supplemental Table 2**.

Supplemental Table 1 Zidovudine and Zidovudine Glucuronide Human PK Reported in Literature

Compound	Dosing Regimen	Subjects				Reference
		Population	No. of Subjects	Age (years)	% Female	
Zidovudine	100 mg SD	HIV positive	24	26-39	36.8%	(Shelton et al., 1994)
	200 mg SD (study day)[a], before study day, 200 mg zidovudine administered orally every 4 h while awake.	HIV positive	11	21-49	0%	(Sahai et al., 1992)
	250 mg (study day)[a], before study day, subjects were treated with zidovudine within 2 months of the diagnosis of AIDS.	HIV positive	13	20-32	23.1%	(Lotterer et al., 1991)
	200 mg TID[a], before study initiation, subjects received 200 mg TID more than 16 weeks.	HIV positive	8	26-41	12.5%	(Gallicano et al., 1999)
	200 mg SD	HIV positive	15	20-40	13.3%	(Wang et al., 1999)
	200 mg SD	HV (HIV seronegative)	6	26-62	0%	(Singlas et al., 1989)
	300 mg BID[b]	HV	18	23-50	0%	(Vourvahis et al., 2013)

BID: twice a day, HV: healthy volunteers, SD: single dose, TID: three times a day

[a]The subjects were treated with zidovudine before study day, and zidovudine PK is already reached to steady state. However, due to an observed short half-life of the drug (1-1.5 h), the simulation was conducted as single dose of the drug.

Supplemental Table 2 Input Parameters of Zidovudine and Zidovudine 5'-*O*-Glucuronide PBPK Model

Parameter	Zidovudine		Zidovudine 5'-*O*-Glucuronide	
	Value	Source	Value	Source
Physicochemical properties				
Molecular weight (g/mol)	267.24	Simcyp	443.37	Simcyp
Log P	0.05	Simcyp	-1.3	Simcyp
Compound type	Monoprotic acid		Diprotic acid	
pKa	9.7	Simcyp	3.5, 9.7	Simcyp
$f_{u,plasma}$	0.8	Simcyp	0.99	Simcyp
B/P	0.91	Simcyp	0.55	Simcyp
Absorption				
Model	ADAM		NA	
fu,gut	1	Assumed		
Peff,man	3.9115	Predicted[a]		
Ptrans,0	25.354	Predicted[a]		
Absorption rate scalar				
Duodenum	5	Optimized	NA	
Jejunum I/Jejunum II	5/5			
Ileum I/Ileum II/Ileum III/Ileum IV	0.5/0.5/0.5/0.5			
Basolateral absorption rate scalar				
Duodenum	5	Optimized	NA	
Jejunum I/Jejunum II	5/5			
Ileum I/Ileum II/Ileum III/Ileum IV	0.5/0.5/0.5/0.5			
Distribution				
Model	Full PBPK Model		Full PBPK Model	
Vss (L/kg)	1.0411 (Method 2)	Simcyp	0.29041 (Method 2)	Simcyp
Kp scalar	2.47	Simcyp	NA	
Elimination				
Enzyme kinetics				
UGT2B7 Vmax (pmol/min/mg protein)	3100	Simcyp	NA	
UGT2B7 Km (μM)	320	Simcyp		
UGT2B7 Liver/Intestine/Kidney scalar	3.27/0.13/1.64	Simcyp		
HLM CL_{int} (μL/min/mg protein)	1.26	Simcyp		
$CL_{int,bile}$ (μL/min/10^6 cells)	NA		0.029	Simcyp
MechKim Model				
$CL_{PD,basal}$ (mL/min/10^6 PTC)	7.514×10^{-5}	Simcyp	NA	
$CL_{PD,apical}$ (mL/min/10^6 PTC)	7.514×10^{-5}	Simcyp		
$fu_{kidney,cell}$	1	Simcyp		
fu_{urine}	1	Simcyp		
OAT1 Jmax (pmol/min/10^6 PTC)	159.3	Simcyp		
OAT1 Km (μM)	45.9	Simcyp		
fu_{inc}	1	Simcyp		
RAF/REF	3	Simcyp		
Kidney apical efflux $CL_{int,T}$ (μL/min/10^6 PTC)	3.47	Simcyp		
fu_{inc}	1	Simcyp		
RAF/REF	3	Simcyp		
CL_R (L/h)	NA		28.2	Simcyp

B/P: blood to plasma, CL_R: renal clearance, OAT: organic anion transporter, PD: passive diffusion, PTC: proximal tubular cells, RAF: relative activity factor, REF: relative expression factor

[a]Predicted using Simcyp Simulator

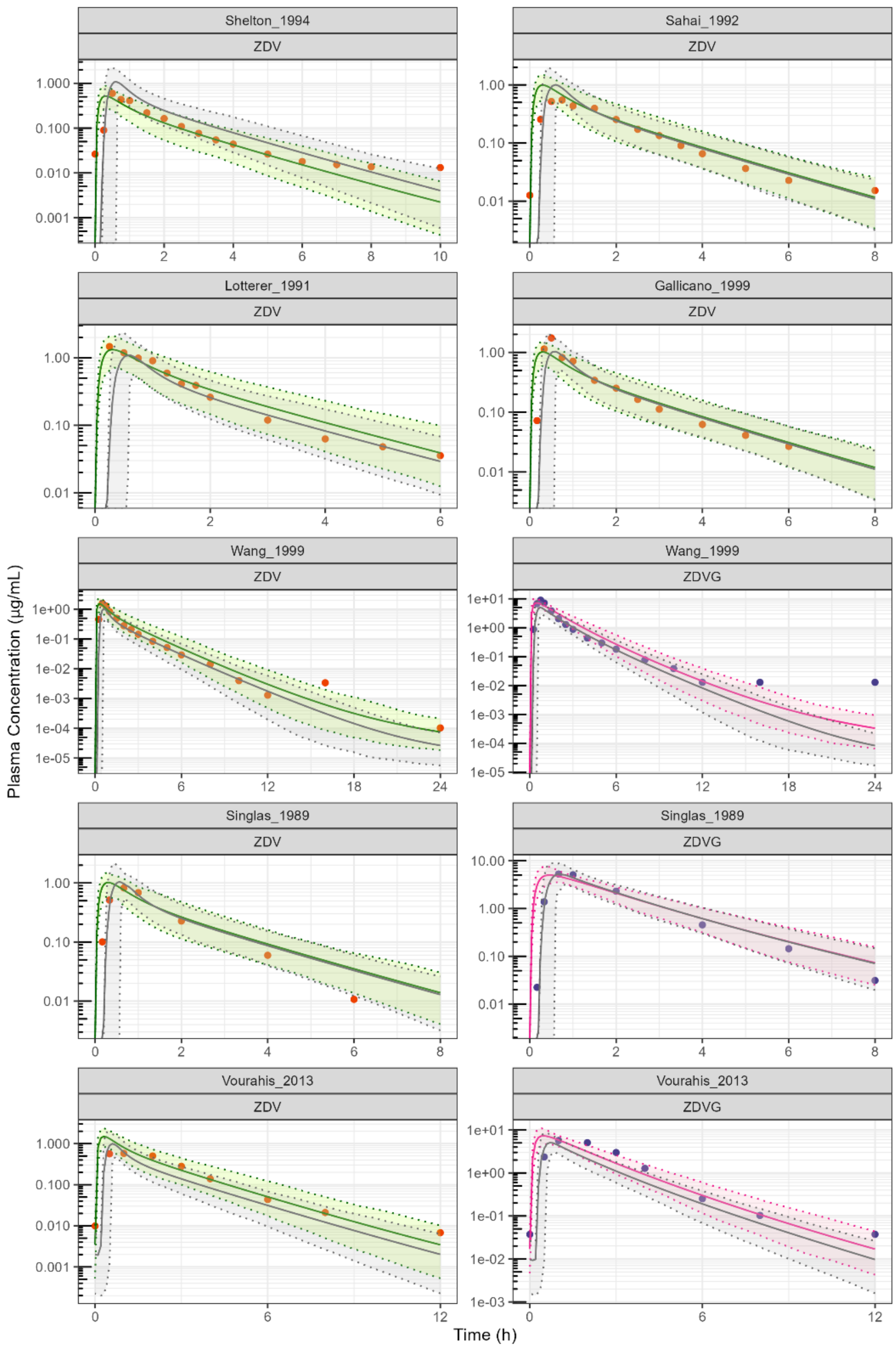

Supplemental Figure 1 Observed Versus Simulated Plasma Profiles of Zidovudine and Zidovudine Glucuronide in Healthy Volunteers or HIV Positive Patients

The simulations were repeated 10 times. The closed orange and dark purple circles represent the observed plasma concentration of zidovudine and zidovudine 5'-*O*-glucuronide, respectively. The gray solid lines represent the zidovudine and zidovudine 5'-*O*-glucuronide mean concentration of virtual population using Simcyp default model. The green and pink solid lines represent the zidovudine and zidovudine 5'-*O*-glucuronide mean concentration of the virtual population using refined model, respectively. The light gray shaded areas represent zidovudine and zidovudine 5'-*O*-

glucuronide 5th-95th percentile of the total virtual population using Simcyp default model. The light green and light pink shaded areas represent zidovudine and zidovudine 5'-*O*-glucuronide 5th-95th percentile of the total virtual population using refined model, respectively.

References

Gallicano KD, Sahai J, Shukla VK, Seguin I, Pakuts A, Kwok D, Foster BC and Cameron DW (1999) Induction of zidovudine glucuronidation and amination pathways by rifampicin in HIV-infected patients. *Br J Clin Pharmacol* **48**:168-179.

Lotterer E, Ruhnke M, Trautmann M, Beyer R and Bauer FE (1991) Decreased and variable systemic availability of zidovudine in patients with AIDS if administered with a meal. *Eur J Clin Pharmacol* **40**:305-308.

Sahai J, Gallicano K, Garber G, McGilveray I, Hawley-Foss N, Turgeon N and Cameron DW (1992) The effect of a protein meal on zidovudine pharmacokinetics in HIV-infected patients. *Br J Clin Pharmacol* **33**:657-660.

Shelton MJ, Portmore A, Blum MR, Sadler BM, Reichman RC and Morse GD (1994) Prolonged, But Not Diminished, Zidovudine Absorption Induced by a High-Fat Breakfast. *Pharmacotherapy: The Journal of Human Pharmacology and Drug Therapy* **14**:671-677.

Singlas E, Pioger JC, Taburet AM, Colin JN and Fillastre JP (1989) Zidovudine disposition in patients with severe renal impairment: influence of hemodialysis. *Clin Pharmacol Ther* **46**:190-197.

Vourvahis M, Davis J, Langdon G, Layton G, Fang J, Choo HW, Hansson AG and Tawadrous M (2013) Pharmacokinetic interactions between lersivirine and zidovudine, tenofovir disoproxil fumarate/emtricitabine and abacavir/lamivudine. *Antivir Ther* **18**:745-754.

Wang LH, Chittick GE and McDowell JA (1999) Single-dose pharmacokinetics and safety of abacavir (1592U89), zidovudine, and lamivudine administered alone and in combination in adults with human immunodeficiency virus infection. *Antimicrob Agents Chemother* **43**:1708-1715.
